# *What price quitting?* The price of cigarettes at which smokers say they would seriously consider trying to quit

**DOI:** 10.1186/1471-2458-13-650

**Published:** 2013-07-13

**Authors:** Michelle Scollo, Linda Hayes, Melanie Wakefield

**Affiliations:** 1 Centre for Behavioural Research in Cancer, Cancer Council Victoria, 1 Rathdowne Street, Carlton, Vic 3053, Australia

**Keywords:** Tobacco excise, Tax, Quit, Smokers, Australia

## Abstract

**Background:**

Deciding on an appropriate level for taxes on tobacco products is a critical issue in tobacco control. The aim of the present study was to describe the critical price points for packs for smokers of each pack size, to calculate what this would equate to in terms of price per stick, and to ascertain whether price points varied by age, socio-economic status and heaviness of smoking.

**Methods:**

In November 2011, 586 Victorian smokers of factory-made cigarettes were asked during a telephone survey about their usual brand, including the size and cost of their usual pack. They were also asked about use of illicit tobacco. Smokers estimated what price their preferred pack would need to reach before they would seriously consider quitting.

**Results:**

Three-quarters of regular smokers of manufactured cigarettes could envisage their usual brand reaching a price at which they would seriously consider quitting. Analyses revealed that answers clustered around whole numbers, (AUD$15, $20, $25 and $30), with a median nominated price point of AUD$20 per pack. The median price point at which regular smokers would consider quitting was calculated to be 80 cents per stick, compared to the current median reported stick price of 60 cents.

Of the smokers who nominated a price point, 60.1% indicated they would seriously consider quitting if the cost of their usual brand equated to 80 cents per stick or less; 87.5% would seriously consider quitting if sticks reached one dollar each.

**Conclusions:**

These results do suggest a potentially useful approach to setting taxes in Australia. If taxes can be set high enough to ensure that the cost of the smokers’ preferred packs exceeds critical price points, then it seems likely that more people would seriously attempt to quit than if the price increased to a level even slightly below the price points. Our study suggests that a tax increase large enough to ensure that a typical pack of 25 cigarettes in Australia cost at least AUD$20 would prompt more than 60% of smokers able to nominate a price point to seriously think about quitting, with particularly strong effects among low-SES smokers.

## Background

Tax increases to reduce demand for tobacco are a crucial component of the comprehensive package of measures needed to reduce the social costs and human toll caused by smoking, [1,2] and form a key plank of the WHO Framework Convention on Tobacco Control. This Convention, adopted by the World Health Assembly on 21 May 2003 and entered into force on 27 February 2005, [3] has become one of the most rapidly and widely embraced treaties in United Nations history with 175 signatories as at June 2012 [4].

Deciding on an appropriate level for taxes on tobacco products is a critical issue in tobacco control. There are several possible approaches.

### Tobacco taxes to recoup social costs

Many economists would argue that the ‘correct’ level of tobacco taxation is that which ensures that revenue from tobacco taxes is at least equal to the costs likely to be imposed on society resulting from tobacco use (that is, those costs not borne voluntarily by smokers themselves) [5]. While theoretically a compelling proposition, in practice governments attempting to set tax levels on this basis face difficulties quantifying future costs resulting from present smoking. Because most health effects occur only after many years of use, and because medical treatments change rapidly, it is extremely difficult to quantify such costs with any accuracy. Another challenge would be to ensure that *all* the relevant costs were included. For many of the health problems caused by smoking, the extent of mortality and morbidity has yet to be quantified [6,7]. It is difficult to imagine how intangible costs such as the pain and suffering of those who lose friends and family members before their time could ever be adequately quantified.

### Tobacco taxes as a corrective force to combat failures of self-control

While the financial and health consequences of smoking might be considered disincentive enough, they largely occur in the future and can be easily dismissed or discounted in a cognitive fashion by smokers. By contrast, the difficulty of quitting must be faced in the present with little prospect of immediate benefit. Increases in the cost of tobacco products help to tip the balance, so that the continuing costs of smoking are greater than the costs of quitting as measured by the immediate level of discomfort. As described by Gruber and Koszegi, governments can provide ‘a self-control device that will allow the consumer to avoid making sub-optimal consumption decisions’ (p13) [8] … that will *make the healthier choice the more attractive choice*.

As part of its report to the Government on recommended reforms to Australia’s tax system, a review panel chaired by the then Treasury secretary Ken Henry, undertook an analysis of Australian tobacco tax rates [9] using a model by Gruber and Koszegi that attempted to acknowledge personal costs to the smoker adjusted to take account of people’s tendency to give more attention to the near-term than to the future [8]. While acknowledging the difficulties of quantifying time preferences and warning that considerable uncertainties surrounded their calculations, they concluded that tobacco excise rates could be increased substantially in Australia. Excise duty on tobacco (25 cents per stick at the time the report was drafted and 35 cents per stick in July 2012) might need to rise to almost 50 cents per stick before those smokers who highly ‘privilege’ the present over the future and who were unaware that they had problems controlling their smoking would seriously attempt to quit. While this is an attractive model, the review panel pointed out a number of limitations. First there are the difficulties of measurement. It is difficult to estimate the value of life-years lost and other harms caused by smoking. It is also difficult to measure the strength of an individual’s ‘current period preference’, that is, the extent to which they are likely to favour a reward in the present compared to the same reward in the future, or—to put it another way—the extent to which a future reward would need to be greater before the person chose it rather than a reward in the present. Second, different groups would vary in their likelihood of suffering future harm—with a low likelihood of harm, for instance, if they only smoked the odd cigarette on an occasional basis—and also in the strength of their current period preferences. And yet the same tax would have to be applied to every purchaser [9].

### Pricing products out of the reach of young people

Another approach to establishing the optimal level of tobacco taxes is to attempt to price tobacco products out of the reach of young people.

The recommended retail price of the leading brand of cigarettes in Australia in April 2012 was AUD$17.15 for a pack of 25 cigarettes [10]. This was less than the cost of purchasing a new release DVD (AUD$19.95 for a single disc, Video Ezy April 2012) and less than half the amount the average 15 year old received in weekly pocket money in 2008 (AUD$37.31 per week), according to Australian Secondary Student Survey on Alcohol and other Drugs unpublished data (Victoria White, personal communication, March 2012). A third approach to taxing tobacco products, then, would be to set tax levels on cigarettes so that a week’s supply would cost more than the average week’s disposable pocket money. This might require the price of cigarettes in Australia to more than double. While it may not be feasible to price young people out of the tobacco market altogether, making cigarettes less affordable to young people is likely to remain an important consideration in setting tax levels.

### Taxing to take tobacco products to smokers’ ‘price point’ for quitting

A less drastic and more pragmatic approach to establishing optimal prices and tax levels on tobacco products might be simply to ask smokers about their intentions to quit at various levels of increases in prices. A tobacco industry-sponsored study commissioned in the mid-1970s [11] found that most people reported that they would continue to smoke after a 5 cent per pack tax increase, but that only 41% would continue with a USD$1 increase. Ross, Powell *et al*[12] found that 6 to 16% of US high school students asked said that they would quit if prices were to increase between 5 cents and USD$4 per pack. Ross, Blecher *et al*[13] investigated expectations about quitting in response to price increases among smokers in the US compared to Australia, the UK and Canada and found that higher starting prices, and greater increases in prices increased the likelihood of a smoker reporting that they expected to quit.

A slight variation on this approach is to ask smokers to indicate how expensive cigarettes would need to be before they would seriously think about quitting. The average increase recorded in responses from Australian smokers who were asked this question in the Victorian Smoking and Health Survey in November 2010 was AUD$6.20 (USD$6.52 as at 1 August, 2012), an increase over prices current at that time of almost 40% [14]. This equated to about 85 cents per stick, or AUD$21.25 (USD$22.34) for a pack of 25 cigarettes. This average, however, was based on that reported by all smokers regardless of whether they smoked cigarettes in packets of 20s, 25s, 30s, 40s or 50s.

Since November 1999, excise and customs duty in Australia is levied as a specific duty on each cigarette, with an equivalent duty per kilogram of tobacco in the case of roll-your-own smoking tobacco and cigars and cigarettes weighing more than 0.8 grams [15]. Prior to 1998, taxes in Australia comprised a weight-based excise/customs duty imposed by the national government combined with a value-based ad valorem fee charged by states. This led to the introduction of lighter-weighing cigarettes sold in progressively larger pack sizes, a uniquely Australian phenomenon, which minimized the duty payable and the wholesale value of packs leading to lower state fees as well, and a substantially lower cost per stick. Large packs have remained on the market in Australia although their use declined after they became much more expensive following the abolition of state fees and the November 1999 excise reforms [16]. The three major companies operating in Australia—British American Tobacco Australia, Philip Morris Australia and Imperial Tobacco Australia— sell a large number of brands across the ‘value’ , ‘mainstream’ and ‘premium’ categories [17]. Most of the major brands are available in a range of pack sizes.

While the excise/customs duty is the main factor determining the price of cigarettes, tobacco companies retain considerable scope to vary the cost of particular brands through cross subsidization between brands and by provision of discounts to retailers. Pack size remains a critical pricing strategy. Selling cigarettes in bulk (in packs of 35s, 40s or 50 or in multi-packs or cartons) allows savings in packaging and continues to provide lower prices per stick. The packaging of cigarettes in small quantities (in the increasingly popular packs of 20s) however provides an upfront purchase price lower than the typical pack of 25s and considerably lower than the very large packs of 40s and 50s, recommended retail prices for which have been well over $20 since a large increase in excise and customs duty in April 2010 [18].

The aim of the present study was to describe the critical price points for packs in 2011 for smokers of each possible pack size, to calculate what this would equate to in terms of price per stick, and to ascertain whether price points varied by age, socio-economic status (SES) and heaviness of smoking.

## Method

The Victorian Smoking and Health Survey is commissioned annually by the Centre for Behavioural Research in Cancer (CBRC). In 2011, telephone interviews were undertaken with a representative sample of adults aged 18 years and over, residing in the state of Victoria. A dual frame design for the survey was adopted, whereby the sample frame was generated by random digit dialling (RDD) to both landline and mobile telephones. 3500 interviews were completed with respondents selected by calling landline telephones and 1000 interviews were completed with respondents contacted by calls to mobile phones. The questions, designed by CBRC, were asked within a broader 14-minute survey of smoking related attitudes and behaviours, conducted during weekends and weeknights between November 2nd and December 5th, 2011. The response rate for the survey was 59%. The survey was approved by the Human Research Ethics Committee of the Cancer Council Victoria (HREC 0018).

A widely accepted question assessing tobacco use [19] was used to determine smoking status. For the purposes of this report, respondents were regarded as regular smokers if they currently smoked manufactured cigarettes on a daily or at least weekly basis. Within the survey, regular smokers of manufactured cigarettes were asked to report on their usual brand of cigarettes; *‘Which is your regular brand of manufactured cigarettes?*’ Smokers who stated a regular brand were then asked to report on the price and size of the pack they usually smoked. *‘How much does a pack of your regular brand usually cost?’* and *‘How many cigarettes per packet are there in the pack size you usually buy?’*

In order to examine the *price point* at which regular smokers would seriously consider quitting, this group were also asked *‘What price would your regular pack need to get to before you would seriously try to quit smoking altogether?*

In a different part of the survey, smokers were also asked about their future intention to quit, *‘Do you think you should quit sometime in the future, or are you happy to smoke for the rest of your life?’* Responses of ‘*Should quit’ , ‘Happy to smoke’* and *‘Don’t know/can’t say’* were recorded. They were also asked about use of illicit unbranded tobacco commonly known in Australia as ‘chop chop’.

This study examines the average amount Victorian smokers of manufactured cigarettes spent on their usual packs in 2011 and the estimated price respondents nominated their usual brand of cigarettes would have to reach before they would seriously consider quitting.

### Statistical analysis

To adjust for any inherent differences between the 2011 dual frame sample and the Victorian population age and gender distributions, the sample was weighted based on 2009 Estimated Resident Population statistics [20]. The dual frame data were also weighted to take into account the relative chance of inclusion in the land line or mobile phone sample frame, as well as chance of selection based on the number of landlines in each household and number of in-scope people per household. Analyses showed that the dual frame sample improved the representation of young adults, males and employed persons in the survey compared to a traditional landline only sample [21].

The average price of an individual cigarette stick in 2011 was calculated by dividing the reported cost of a smoker’s usual pack by the number of sticks in the pack. The estimated price an individual stick would have to reach before the smoker would seriously consider quitting was calculated by dividing the price a smoker nominated their usual pack would have to reach before they would seriously consider quitting, by the size of the pack.

Smokers were classified as being light smokers if they smoked fewer than 10 cigarettes on average per day, medium smokers reported smoking between 10 and 19 cigarettes a day and those who smoked 20 cigarettes or more a day were classified as heavy smokers.

A frequency of consumption variable categorised respondents as being daily or weekly (at least weekly, but not daily) smokers.

The Socio-Economic Index for Areas (SEIFA), developed by the ABS, was used to classify respondents into socio-economic groups based on 2006 Census data of the area in which they live [22]. In the following analyses, the Index of Socio-Economic Disadvantage (one of the four ABS SEIFA indexes) has been used, based on respondent’s residential postcode. In Australia area measures of disadvantage are generally considered more reliable than individual measures such as income which suffers from problems of instability over life-course and poor response rates [23].

This index ranks postcodes on a continuum of high disadvantage to low disadvantage, taking into consideration characteristics such as income, education, occupation and housing that may reduce socio-economic conditions of the area. For the purpose of analysis we have aggregated respondents into three groups based on this scale.

The low SES group (1st & 2nd quintiles) comprised people who live in areas with a SEIFA score in the bottom 40% of ranked Victorian postal areas (this represents a higher level of disadvantage relative to the other 2 groups). The mid SES group (3rd & 4th quintiles) includes people who live in areas with a SEIFA score between 41% and 80% of ranked postal areas. The high SES group (5th quintile) includes those who live in areas with a SEIFA score between 81% and 100% of ranked postal areas (reflecting a lower level of disadvantage relative to the other groups). In 2011, just over 32% of the dual frame sample fell into the low SES group (1st and 2nd quintiles); 41% in the mid SES group (3rd and 4th quintiles); and 27% in the high SES group (5th quintile), similar to the Victorian population overall [20].

Analyses of co-variance (ANCOVA) were undertaken to examine the effects of pack size, frequency of smoking and SES on the price individual sticks would have to reach before smokers would seriously consider quitting, taking into account sex, age and consumption levels.

Multivariate logistic regression analyses were undertaken to explore the characteristics of groups who did not (or who were unable to) nominate a price at which they would seriously consider quitting.

## Results

In 2011, 15.0% (n =675) of Victorian adults identified as being regular (daily or weekly) smokers of tobacco products, 86.9% (n=586) of whom were regular smokers of factory-made cigarettes.

### Price point per pack

**Figure 1:**
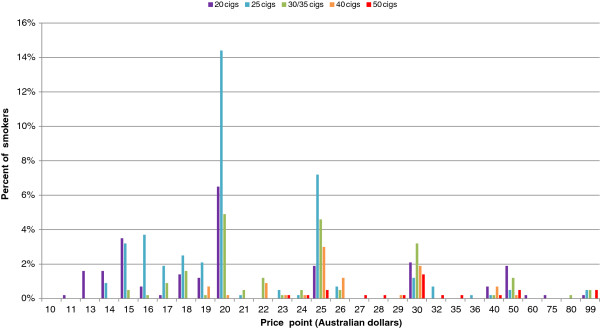
Nominated price point cigarette packs would have to reach before smokers would seriously consider quitting, by current pack size, as a percentage of all regular smokers of manufactured cigarettes.

All regular smokers of manufactured cigarettes identified a brand which they usually smoked. The nominated prices at which respondents would consider quitting ranged from AUD$11 to AUD$99 per pack (Figure 1). The median price of smokers’ usual pack of cigarettes in 2011 was AUD$16 (median pack size of 25). When asked how much the price of their usual pack would have to reach before they would seriously consider quitting, 25.6% (n=150) of regular smokers of manufactured cigarettes did not, or were unable to state a price point, so this group was excluded from further analyses relating to quitting price points. Among the remaining respondents, the typical smoker stated that their pack price would have to increase to AUD$20 dollars before they would seriously consider quitting. Twenty dollars was the most common response for smokers of packs of 20s (25%) 25s (33%) and 30s (25%). Prices paid by smokers for packs of 40s and 50s were already well in excess of $20 at the time of the survey. Table 1 shows that the mean and median price points nominated by smokers of packs of 25s were no higher than those nominated by smokers of packs of 20s.

**Table 1:** Current cost of usual pack of cigarettes and nominated price point usual pack would have to reach before smoker would seriously consider quitting, Victorian adults 2011 dual frame sample

		**Current cost of usual pack (AUD $)**^**1**^		**Nominated quitting price point (AUD $)**		**% increase in median price**
	**Number**	**Mean (SD)**	**Median**	**Mean (SD)**	**Median**	
Total sample	429	16.54 (3.9)	16.00	23.49 (8.9)	20.00	25.0
*Pack size*						
20	102	13.41 ( 2.6)	13.00	22.89 (11.6)	20.00	53.8
25	176	15.62 (1.9)	16.00	21.02 (5.6)	20.00	25.0
30 or 35	88	17.38 (2.1)	17.38	24.97 (9.1)	23.57	35.6
40	41	21.93 (2.2)	22.00	27.07 (6.1)	25.00	13.6
50	17	24.33 (2.2)	25.00	31.54 (7.6)	30.00	20.0
*Level of Consumption*						
Light (<10)	200	15.76 (3.3)	16.00	23.02(7.8)	20.00	25.0
Medium (10 to <20)	146	16.74 (3.6)	16.47	22.84 (9.4)	20.00	21.4
Heavy (20 plus)	79	18.03 (5.0)	17.00	25.61 (10.5)	23.00	35.3
*Frequency of Consumption*						
Daily	360	16.46 (3.5)	16.0	22.84 (8.4)	20.00	25.0
Weekly	69	16.93 (5.2)	17.0	26.84 (10.6)	25.00	47.1
*Quit intention*						
Happy to smoke	23	17.24 (3.8)	16.50	26.58 (7.8)	25.41	54.0
Not happy to smoke	407	16.50 (3.9)	16.00	23.32 (9.0)	20.00	25.0
*Sex*						
Male	223	16.57 (4.1)	16.00	24.02 (9.5)	20.00	25.0
Female	206	16.50 (3.6)	16.00	22.91 (8.2)	20.00	25.0
*Age*						
18 to 29 years	136	16.14 (3.7)	16.00	23.33 (8.0)	20.00	25.0
30 to 49 years	202	16.57 (4.0)	16.20	23.39 (8.8)	20.00	23.5
50 plus	91	17.05 (3.9)	16.00	23.96 (10.5)	20.00	25.0
*SES*						
Low	163	17.10 (4.0)	16.59	23.42 (9.0)	20.00	20.6
Medium	179	16.21 (3.3)	16.00	22.53 (7.8)	20.00	25.0
High	88	16.16 (4.6)	16.00	25.56 (10.6)	20.00	25.0

Footnote: excludes those who did not nominate a price point (n=150 of regular smokers of manufactured cigarettes)^. 1^AUD$1 = USD$1.05 as at 1 August 2012.

When comparing responses by pack size, the largest proportional increase in the median pack price necessary to promote quitting, was reported by smokers with a usual pack size of 20 cigarettes (Table 1). The median price of a 20 cigarette pack in 2011 was AUD$13, and the typical smoker of a 20 pack stated that the price of their pack would have to reach AUD$20 (an increase of more than 50%) before they would seriously consider quitting. The lowest percentage increase in median pack price was nominated by smokers of packs containing 40 cigarettes. This group estimated an increase in price of 13.6% (from the current median price (AUD$22) to the median nominated price point (AUD$25)) would be necessary before they would seriously consider quitting.

For all key demographic groups, AUD$20 was the median price that packs would have to reach before regular smokers would seriously consider quitting.

In 2011, only one in ten (9.6%) of all regular smokers of factory made cigarettes reported that they would be happy to smoke for the rest of their lives. Among those who were happy to continue smoking and who nominated a price point, the median current price of their usual pack was AUD$16.50 and the median quitting price point nominated was AUD$25.41 (a potential increase of more than 50%).

The nominated price points at which smokers would seriously consider quitting tended to cluster around particular numbers, with smokers of all pack sizes represented at almost every $5 multiple. Approximately one quarter (26.2%) of smokers who gave an estimated price point, reported that their usual pack would have to reach AUD$20 before they would seriously consider quitting, 17.2% nominated AUD$25 as being the point at which they would consider quitting, while 9.9% cited AUD$30 as being the price point that their pack would have to reach before they would seriously consider quitting. Unsurprisingly, users of large packs tended to cluster at higher price points than users of smaller packs. The average percentage increase over the current price before smokers would seriously consider quitting was 18% for those smoking pack sizes of 35 or greater but almost 54% for those smoking packs of 20.

### Price point per stick

The mean and median cost of individual cigarettes and the mean and median price points individual cigarettes would have to reach before smokers would seriously consider quitting are presented in Table 2. As shown, the median price point at which regular smokers would consider quitting was calculated to be 80 cents per stick, compared to the current median reported stick price of 60 cents.

**Table 2:** Current cost of individual sticks from usual pack of cigarettes and nominated price point individual sticks would have to reach before smokers would seriously consider quitting, Victorian adults 2011 dual frame sample

		**Current cost of stick from usual pack AUD $**		**Nominated quitting price point, per stick AUD $**		**% increase in median price**
	**Number**	**Mean (SD)**	**Median $**	**Mean (SD)**	**Median $**	
Total sample	425	0.61 (0.10)	0.60	0.89 (0.39)	0.80	33.3
*Pack size*						
20	102	0.67 (0.13)	0.65	1.14 (0.58)	1.00	53.8
25	176	0.62 (0.08)	0.64	0.84 (0.22)	0.80	25.0
30 or 35	88	0.58 (0.07)	0.58	0.83 (0.31)	0.79	36.2
40	41	0.55 (0.05)	0.55	0.68 (0.15)	0.63	14.5
50	17	0.49 (0.04)	0.50	0.63 (0.15)	0.60	20.0
*Level of Consumption*						
Light (<10)	199	0.62 (0.11)	0.60	0.92 (0.35)	0.80	33.3
Medium (10 to <20)	146	0.61 (0.08)	0.62	0.85 (0.39)	0.78	37.1
Heavy (20 plus)	75	0.60 (0.08)	0.60	0.88 (0.46)	0.75	46.7
*Frequency of consumption*						
Daily	359	0.61 (0.08)	0.60	0.86 (0.37)	0.80	33.3
Weekly	65	0.64 (0.16)	0.64	1.04 (0.44)	1.00	56.3
*Quit intention*						
Happy to smoke	23	0.57 (0.10)	0.55	0.90 (0.32)	0.88	60.0
Not happy to smoke	402	0.62 (0.10)	0.61	0.89 (0.39)	0.80	31.0
*Demographics*						
*Sex*						
Male	219	0.61 (0.10)	0.61	0.91 (0.41)	0.80	31.1
Female	206	0.61 (0.10)	0.60	0.87 (0.36)	0.80	33.3
*Age*						
18 to 29 years	136	0.62 (0.12)	0.62	0.91 (0.36)	0.80	29.0
30 to 49 years	198	0.62 (0.09)	0.62	0.89 (0.39)	0.80	29.0
50 plus	90	0.59 (0.09)	0.59	0.84 (0.42)	0.72	22.0
*SES*						
Low	161	0.62 (0.11)	0.60	0.87 (0.40)	0.78	30.0
Medium	179	0.61 (0.08)	0.60	0.86 (0.33)	0.80	33.3
High	85	0.61 (0.12)	0.63	0.99 (0.44)	0.95	50.8
Purchased unbranded tobacco in last 12 months						
Yes	14	0.65 (0.10)	0.67	1.40 (0.60)	1.43	>100
No	411	0.61 (0.10)	0.60	0.87 (0.36)	0.80	33.3

Footnote : excludes respondents who did not nominate a price point (n=150 of regular smokers of manufactured cigarettes) and respondents whose pack size was unknown. Stick prices are calculated based on reported pack prices and sizes.

**Figure 2:**
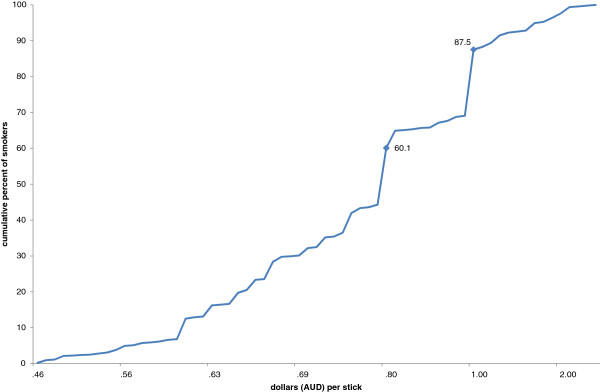
**Cumulative per cent of smokers who stated that they would seriously consider quitting, by price point, 2011—expressed as cents per stick.** Footnote: Excludes the 150 respondents who did not nominate a price point.

Over half of smokers (60.1%) who nominated a price point, indicated a price point for their usual brand at which they would seriously consider quitting of an amount that equated to 80 cents per stick or less (Figure 2). The total percentage of people who report that they would seriously consider quitting rose steadily for price points equating to between 60 and 79 cents per stick, and jumped sharply at 80 cents per stick and then again at $1 per stick. The majority of smokers who nominated a price point (87.5%) would seriously consider quitting if each stick within their usual pack cost one dollar.

Fewer than 3.6% of smokers reported having used illicit tobacco at least once over the previous 12 months. Findings of another very large Australian survey [24] suggest that the majority of these smokers would no longer be using this type of tobacco or would use it only occasionally. The proportion of Australian smokers who reported using unbranded tobacco ‘about half the time or more’ in 2010 was less than 1.5% [24].

The 14 smokers who had both used illicit tobacco sometime in the past twelve months and were able to nominate a price point, reported that cigarettes would need to increase in price by an average of 100% before they would seriously consider quitting. Numbers were too small to allow inclusion of this factor in multivariate analysis.

ANCOVA was used to explore the adjusted effect of pack size, frequency of smoking and SES on the nominated price point individual sticks would have to reach before smokers would consider quitting (Table 3). After adjusting for sex, age group, and consumption levels, no significant differences were found in the average price points nominated by smokers from the low (most disadvantaged), medium and high (least disadvantaged) SES groups, although there was evidence of a trend for higher SES respondents to report a higher price point.

**Table 3:** Nominated price point individual sticks would have to reach before smokers would seriously consider quitting by pack size and SES, Victorian adults 2011 dual frame sample (ANCOVA)

	**Number**	**Estimated marginal means (SE)**	**F (df)**	**p-value**
*Pack size*			19.524 (4)	0.000
20	100	1.21 (0.04)		
25	171	0.90 (0.03)		
30 or 35	89	0.91 (0.05)		
40	42	0.71 (0.06)		
50	15	0.72 (0.10)		
*SES*			2.428 (2)	0.089
Low	158	0.87 (0.04)		
Medium	180	0.85 (0.04)		
High	79	0.96 (0.05)		
*Frequency of consumption*			6.834 (1)	0.009
Daily	352	0.82 (0.03)		
At least weekly	65	0.96 (0.05)		

Note: Model adjusted for sex, age group, and level of consumption . Excludes respondents who did not nominate a price point.

Pack size was found to be independently related to the price point at which smokers reported they would seriously consider quitting. Smokers of smaller packs reported that higher stick prices would be necessary before they would seriously consider quitting. On average, after adjusting for sex, age and consumption levels, smokers of packs containing 20 cigarettes nominated price points that indicated that sticks in their usual pack would have to reach AUD$1.21 each before they would consider quitting, a significantly higher per stick price than the average price points nominated by smokers of each of the larger pack sizes. Frequency of cigarette consumption was also found to be independently associated with per stick price point, with smokers smoking at least weekly but less-than daily nominating significantly higher stick price points (Estimated Marginal Mean=AUD$ 0.96, SE=0.05) compared to daily smokers (Estimated Marginal Mean=AUD $0.82, SE=0.03).

### Characteristics of those who did not nominate a quitting price point

Multivariate logistic regression analyses, adjusting for all variables in the model were undertaken to explore the characteristics of the 25.6% of smokers (n=150) who did not nominate a price point at which they would seriously consider quitting (Table 4). The strongest single predictor of an individual not nominating a price point was their future intention to quit. Respondents who stated that they were happy to smoke for the rest of their lives had approximately three times the odds of not nominating a price point, compared to those who reported that they were not happy to continue smoking. Just over half of smokers (53.4%) who were happy to continue smoking did not state a quitting price point, compared to 22.7% of those who were not happy to smoke for the rest of their lives.

**Table 4:** Characteristics associated with not nominating a price point, adjusting for all variables in model

	**Number**	**% unable to nominate a price point**	**Adjusted odds ratio**^**#**^	**95% Confidence interval**	**P - value**
Total sample	586	25.6			
*Pack size*					
20	121	14.2	1.00		
25	240	26.0	1.87	1.00-3.48	0.049
30 or 35	118	23.4	1.27	0.62-2.60	0.516
40	70	41.3	2.53	1.17-5.47	0.019
50	28	32.6	1.47	0.51-4.21	0.478
*Level of Consumption*					
Light	250	19.4	1.00		
Medium	188	21.8	0.74	0.44-1.26	0.268
Heavy	144	41.8	2.13	1.25- 3.62	0.005
*Frequency of Consumption*					
Daily	504	27.5	1.00		
Weekly	82	14.2	0.38	0.16-0.89	0.025
*Quit intention*					
Not happy to smoke	530	22.7	1.00		
Happy to smoke	56	53.4	3.47	1.86-6.47	0.000
*Demographics*					
*Sex*					
Male	307	26.4	1.00		
Female	279	24.8	1.12	0.73-1.71	0.616
*Age*					
18 to 29 years	163	14.5	1.00		
30 to 49 years	274	25.8	1.97	1.13-3.43	0.017
50 plus	150	37.5	2.84	1.55-5.22	0.001
*SES*					
Low	222	25.3	1.00		
Medium	252	27.8	1.30	0.83-2.06	0.253
High	112	21.6	1.07	0.59-1.97	0.817

Footnote= 1.00 reference category for logistic regression analyses, # multivariate model adjusting for all variables.

Daily smokers were significantly less likely to nominate a price point when compared to weekly smokers. 41.8% of heavy smokers did not state a price point compared to 19.4% of light smokers, a significant difference. No difference was found when comparing light smokers’ and medium smokers’ likelihood of not nominating a price point. After adjusting for the other variables in the model, smokers of packs of 25 and 40 cigarettes were more likely than smokers of packs containing 20 cigarettes to not nominate a price point. 41.3% of those who smoked 40 packs and 26.0% of smokers of 25 packs, did not nominate a price point compared to 14.2% of adults who regularly smoked cigarettes from the smallest packs. No differences were found when comparing smokers of 30/35 or 50 packs tendency to nominate price points, with smokers of the smallest packs.

Of the demographic characteristics studied, sex and SES were not associated with likelihood of nominating a quitting price point. However, compared to younger adults (14.5%), adults aged 50 plus (37.5%) and 30 to 49 years (25.8%) were more likely to be represented in the respondents who did not nominate price points. Additional analysis explored the relationship between ability to nominate a price point and use of illicit (unbranded tobacco). Only 21 people indicated that they had purchased such tobacco at least once in last 12 months. The percentage of these unable to nominate a price point (28.1%) was not significantly higher than the percentage unable to nominate a price point among those who had made no such purchases (25.6%).

## Discussion

The results of this analysis suggest that three quarters of smokers can envisage a price for their usual brand of cigarettes beyond which they would seriously think about trying to stop smoking. This of course does not mean that all these smokers would actually attempt to quit were such prices to eventuate or that the majority of them would succeed. Economists have long known that participants in ‘willingness to pay’ studies are subject to ‘hypothetical bias’, tending to exaggerate the amounts they would pay for social goods (such as saving a particular endangered animal species etc.) [25] In the absence of actual market data (on people’s payments for such goods in the real world), environmental research experts recommend dividing hypothetical payments by two [26]. It is unclear how hypothetical bias might be corrected in the case of an addictive product such as tobacco. This is something that could be investigated in cohort studies of smokers faced with hypothetical and then with real price increases over the course of several years. A substantial minority of respondents in the study—about a quarter—were unable to answer the question as posed. The reasons for this were not explored in detail in this study. It is not known how many respondents were not confident they could quit even with a price increase, and how many for whom price is simply not a factor. However it is reassuring that smokers unable to nominate a price point were no more likely than those able to nominate a price point to be of low-SES.

Rounding effects would appear to be extremely important in tobacco pricing. The AUD$20 packet of cigarettes is likely to be a trigger to quit not just for smokers of packs of 20s but also for a great many smokers of packs of 25s and 30s. Packs of 35s, 40s and 50s already exceed this price and the AUD$25 and AUD$30 pack would seem to be the price points for many 35/40s and 50s smokers respectively. Tobacco companies are well aware of price point phenomena [27]. Multiple pack sizes and the capacity to increase profit margins on some brands in order to maintain price sensitive customers of other brands [28] allows companies to keep the prices of each brand under critical price points (currently $20, $25 and $30).

The trade-off in the price *per stick* and the price *per pack* evident in the Australian market creates an interesting dynamic with regard to efficiency of tax increases. Use of packs of 20s has increased quite substantially in Australia over the past five years, no doubt driven by the substantially lower up-front purchase price than that which applies to packets of 30s, 35s, 40s and 50s [16]. It appears that encouraging smokers of cigarette brands sold in packs of 20s to quit will necessitate much larger increases in duty than would be the case for smokers of packs of 30s and 40s.

This study illustrates one mechanism which helps to explain why low-SES smokers are usually found to be more price sensitive than high-SES smokers. At 87 cents per stick, the typical low-SES smoker would seriously think about quitting, while for the typical high-SES that figure is more like 96 cents per stick. It also suggests a strategy for optimising quitting among low-SES groups while at the same time minimising the additional financial outlay required by continuing smokers. More than 60% of smokers able to nominate a price point would seriously think about quitting if retail prices of cigarettes were at least 80 cents per stick. Almost nine out of ten of the 74% of smokers who were able to indicate a price point would seriously think about quitting if their cigarettes cost at least $1 per stick. Crucially however, the results suggest that little additional benefit in terms of quitting behaviour and public health consequences would be provided by prices greater than 80 cents until the AUD$1 threshold is reached.

## Conclusions

The study provides guidance for policy-makers contemplating increases in excise/customs duty on tobacco products, showing both the limits and the potential value of price as a strategy to increase quit rates.

Companies faced with increases in excise and customs duty are still able to cushion smokers against the effects of duty increases by increasing margins on some brands in order to cover lower margins on other brands that might be attractive to price sensitive smokers. Smokers can also minimise the effects of tax increases by seeking out retailers that provide tobacco products at discounted rates and by shifting to smaller pack sizes. The ability of manufacturers to keep prices under critical price points greatly reduces the impact of increases in excise and customs duty.

More than a quarter of all smokers are unable to envisage a price at which they would seriously consider quitting smoking, and for ten per cent of smokers the price point would be almost AUD$50 per pack. Price points were also very high for the small number of smokers who had used illicit tobacco. Ensuring that prices of all cigarettes were high enough to encourage the bulk of smokers to think about quitting would necessitate very large increases in taxes and prices—price increases of up to 50% per pack (more than double the large increase in prices resulting from the most recent increase in excise/customs duty in April 2010). While many smokers would quit, increases of this magnitude would entail additional spending on tobacco products that many smokers who do not quit may find difficult to accommodate. Governments may also be concerned that tobacco prices extremely high relative to those of neighbouring countries would increases incentives for tax evasion and growth in the market for illicit tobacco. These concerns underscore the need for a range of complementary non-price regulatory and educational measures to outlaw the promotion of tobacco products and to educate smokers about the risks of smoking and medicinal aids and behavioural tips to improve success rates in quitting. The results of this study do however suggest a potentially useful approach to setting taxes in Australia and perhaps in other countries. If taxes can be set high enough to ensure that the cost of the smokers’ preferred packs exceeds critical price points, then it seems likely that more people would seriously attempt to quit than if the price increased to a level even slightly below the price points. Our study suggests the need for a tax increase large enough to ensure that a typical pack of 25 cigarettes in Australia cost at least AUD$20 in most retail outlets. An increase of this magnitude would prompt more than 60% of smokers able to nominate a price point to seriously think about quitting, with particularly strong effects among low- and medium-SES smokers.

## Competing interests

The authors declare that they have no competing interests.

## Authors’ contributions

MS conceived the study with help from MW, and LH oversaw fieldwork and did the analysis under the supervision of MW. All authors contributed to the writing of the manuscript and approved the final version for submission.

## Pre-publication history

The pre-publication history for this paper can be accessed here:

http://www.biomedcentral.com/1471-2458/13/650/prepub
